# Characterization of Toxigenic *Vibrio cholerae* from Haiti, 2010–2011

**DOI:** 10.3201/eid1711.110805

**Published:** 2011-11

**Authors:** Deborah Talkington, Cheryl Bopp, Cheryl Tarr, Michele B. Parsons, Georges Dahourou, Molly Freeman, Kevin Joyce, Maryann Turnsek, Nancy Garrett, Michael Humphrys, Gerardo Gomez, Steven Stroika, Jacques Boncy, Benjamin Ochieng, Joseph Oundo, John Klena, Anthony Smith, Karen Keddy, Peter Gerner-Smidt

**Affiliations:** Centers for Disease Control and Prevention, Atlanta, Georgia, USA (D. Talkington, C. Bopp, C. Tarr, M.B. Parsons, M. Freeman, K. Joyce, M. Turnsek, N. Garrett, M. Humphrys, G. Gomez, S. Stroika, P. Gerner-Smidt); Centers for Disease Control and Prevention, Port-au-Prince, Haiti (G. Dahourou); CDC–Kenya Medical Research Institute, Kisumu, Kenya (B. Ochieng, J. Oundo); Chinese Center for Disease Control and Prevention, Beijing, People’s Republic of China (J. Klena); Ministry of Public Health and Population, Port-au-Prince (J. Boncy); National Institute for Communicable Diseases, Johannesburg, South Africa (A. Smith, K. Keddy)

**Keywords:** Vibrio cholerae, Vibrio cholerae O1, bacteria, disease outbreaks, Haiti, public health, diarrhea, cholera, cholera toxin, epidemics

## Abstract

In October 2010, the US Centers for Disease Control and Prevention received reports of cases of severe watery diarrhea in Haiti. The cause was confirmed to be toxigenic *Vibrio cholerae*, serogroup O1, serotype Ogawa, biotype El Tor. We characterized 122 isolates from Haiti and compared them with isolates from other countries. Antimicrobial drug susceptibility was tested by disk diffusion and broth microdilution. Analyses included identification of *rstR* and VC2346 genes, sequencing of *ctxAB* and *tcpA* genes, and pulsed-field gel electrophoresis with *Sfi*I and *Not*I enzymes. All isolates were susceptible to doxycycline and azithromycin. One pulsed-field gel electrophoresis pattern predominated, and *ctxB* sequence of all isolates matched the B-7 allele. We identified the *tcpET*^CIRS^ allele, which is also present in Bangladesh strain CIRS 101. These data show that the isolates from Haiti are clonally and genetically similar to isolates originating in Africa and southern Asia and that *ctxB*-7 and *tcpET*^CIRS^ alleles are undergoing global dissemination.

## Abstract 2

A virulent clone from Africa or southern Asia was likely introduced at a single time point.

*Vibrio cholerae* has caused epidemics around the world for centuries. Cholera has long been a companion of devastation and poverty, and epidemics occur in areas without clean water, sanitation, or accessible health care. The collapse of Haiti’s infrastructure after the January 2010 earthquake created conditions suitable for cholera to affect the country’s vulnerable population.

The first clinical cases of *V. cholerae* infection in Haiti in >100 years were seen on October 17, 2010. Cholera, primarily a waterborne disease, quickly spread from its origin along a main river in the Artibonite Department north of Port-au-Prince to all 10 departments in Haiti and to the Dominican Republic. Earlier that year, in anticipation of outbreaks after the devastation of the January 2010 earthquake, the Centers for Disease Control and Prevention (CDC; Atlanta, GA, USA) had collaborated with local scientists to tactically position rapid diagnostic tests and had provided training in their use. These tests enabled early recognition of the 2010 cholera outbreak in Haiti. By October 21, 2010, scientists at the Haiti National Public Laboratory (Laboratoire National de Santé Publique) cultured *V. cholerae* and sent isolates to CDC (*1*).

Our objectives were to confirm the identification of *V. cholerae*, characterize the isolates by using multiple genetic and phenotypic methods, evaluate the clonality of the isolates from Haiti, and attempt to explore the genetic origin of the strain. Isolates from Haiti were compared with isolates from western and eastern Africa, southern Asia, Latin America, the Middle East, and the Gulf Coast of the United States.

## Methods

### Bacterial Strains

A total of 122 *V. cholerae* isolates from all 10 departments in Haiti were characterized. We also included 25 *V. cholerae* isolates that showed hemolytic and nonhemolytic phenotypes on sheep blood agar and 2 chloramphenicol-resistant colonies that grew inside the zone of inhibition, for a total of 149 isolates from Haiti. The isolates arrived in 4 groups that were received on October 26, 2010 (16 isolates), November 26, 2010 (92 isolates), January 27, 2011 (30 isolates), and February 9, 2011 (11 isolates). We also studied 51 *V. cholerae* strains from other countries; they were obtained during past outbreaks and from sporadic cases and ongoing CDC surveillance of travelers entering the United States. Additional strains were provided by collaborators at CDC-Kenya Medical Research Institute (Kisumu, Kenya); Naval Medical Research Unit 3 (Cairo, Egypt); and the National Institute of Communicable Diseases (Johannesburg, South Africa). The origins of the strains evaluated in this study are shown in Table 1. Isolate CDC 2010EL-1786 from Haiti was deposited in the American Type Culture Collection (ATCC; BAA-2163).

**Table 1:** Strains used in characterization study of *Vibrio cholerae* from Haiti, 2010–2011*

Location	No. isolates	Year(s) collected
Afghanistan	2	2008
Cameroon	1	2010
Djibouti	2	2007
Ethiopia	1	2009
Haiti	149	2010–2011
India	6	2005–2009
Kenya	14	2007–2009
Nepal	1	2008
Nigeria	2	2008
Oman	1	2007
Pakistan	6	2005–2010
Peru	2	1991, 1998
Somalia	2	2008
South Africa	2	2009
Sri Lanka	1	2007
Sudan	5	2007
Togo	2	2009
US Gulf Coast	1	2007
Total	200	

*Isolates collected during 1991–2011.

### Confirmation and Characterization of *V. cholerae*

All isolates were confirmed positive by using standard methods (*2*). Serogroup and serotype were determined by using specific antisera (Lee Laboratories, Franklin Lakes, NJ, USA). Saline controls were included to detect autoagglutination. Biotypes were determined by PCR of specific regions of the biotyping (*tcpA*) gene (*3*).

### Antimicrobial Drug–Susceptibility Testing

Pure cultures were tested by disk diffusion on Mueller-Hinton agar without blood with amoxicillin/clavulanate, ampicillin, chloramphenicol, ciprofloxacin, furazolidone, nalidixic acid, streptomycin, sulfisoxazole, tetracycline (as a marker for doxycycline), and trimethoprim/sulfamethoxazole (Becton Dickinson, Franklin Lakes, NJ, USA). ATCC (Manassas, VA, USA) strains 25922 (*Escherichia coli*), 29213 (*Staphylococcus aureus*), and 27853 (*Pseudomonas aeruginosa*) served as internal quality controls. Results were interpreted according to Clinical and Laboratory Standards Institute guidelines (*4*).

Broth microdilution was run on 122 isolates from Haiti and on all isolates from non-Haiti locations. Broth microdilution methods were performed according to the manufacturer’s instructions by using CAMPY and CMV1AGNF Sensititer Plates (both from Trek Diagnostics, Cleveland, OH, USA) with 2 modifications: we transferred 50-μL (CAMPY plates) and 20-μL (Sensititer plates) volumes from the suspension to the broth and used Mueller-Hinton broth without blood for the CAMPY panel. Antimicrobial drug sensitivity results from Sensititer plate testing were available for amoxicillin/clavulanate, ampicillin, azithromycin, chloramphenicol, ciprofloxacin, nalidixic acid, streptomycin, sulfisoxazole, tetracycline, and trimethoprim/sulfamethoxazole. Both panels were inoculated at concentrations of 5 × 10^4^ and 5 × 10^5^ CFU/mL. Internal quality controls included those used for disk-diffusion testing plus ATCC 29212 (*Enterococcus faecalis*). Where available, specific interpretive criteria for *V. cholerae* were used (*5*). For drugs with no criteria, interpretation was guided by using Clinical and Laboratory Standards Institute criteria for *Enterobacteriaceae* or consensus breakpoints used by the National Antimicrobial Resistance Monitoring System (*6*).

### Pulsed-Field Gel Electrophoresis

Isolates were analyzed by using a PulseNet standardized pulsed-field gel electrophoresis (PFGE) protocol for *V. cholerae* (*7*) with *Sfi*I and *Not*I restriction enzymes (Roche Molecular Biochemicals, Indianapolis, IN, USA). Images of restriction patterns were analyzed by using BioNumerics software (Applied Maths, Inc., Austin, TX, USA). Gel patterns were compared with others in the National PulseNet *V. cholerae* database (http://www.cdc.gov/pulsenet/whatis.htm) and the PulseNet International *V. cholerae* database (www.pulsenetinternational.org/protocols/Pages/vcholeraedatabase.aspx).

### Detection of Virulence and Species-specific Genes, PCR, and Sequencing

We amplified DNA from boiled lysates for 30 cycles in a multiplex PCR to detect cholera toxin gene subunit A (*ctxA*) (*8*) sequences, biotyping genes (*tcpA*) (*3*), and species-specific genes *ompW* (*9*), and *toxR* (*10*) by using the primers and methods described. Primer pair *smp-*F and *smp*-R (*11*) was used to amplify the seventh pandemic–specific gene VC2346 at cycling conditions of 93°C for 15 min; 35 cycles of 92°C for 40 s, 52°C for 1 min, and 72°C for 1.5 min; followed by 72°C for 7 min.

The primers and cycling conditions for PCR amplification of biotype-specific repeat sequence transcriptional regulator (*rstR*) alleles were used as described (*12*). We sequenced 13 isolates from Haiti and from the strain from the US Gulf Coast by using the same primer set to verify results.

The complete coding region of the cholera toxin gene *ctxAB* was amplified with flanking primer pair primers S86 (*ctxAB*_1_) and S87 (*ctxAB*_2_) (*13**,**14*). A step-down PCR to avoid nonspecific amplification was run as follows: 15 min at 93°C; 11 cycles of 92°C for 40 s, decrementing by 1°C from 60°C to 50°C for 1 min, 72°C for 1.5 min; followed by 30 cycles of 92°C for 40 s, 50°C for 1 min, 72°C for 1.5 min; with a final extension at 72°C for 7 min. Sequences were determined with amplification primers and 2 internal primers, CTX93-F and CTX618-R (*15*). The complete *tcpA* gene was amplified and sequenced with primer pair *tcp*H1 and *tcpA*4 (*16*). PCR cycling conditions were 93°C for 15 min followed by 35 cycles of 92°C for 40 s, 52°C for 1 min, and 72°C for 1.5 min, with a final extension at 72°C for 7 min.

We purified *ctxAB*, *tcpA*, and *rstR* amplicons by using the QIAquick PCR Purification Kit (QIAGEN, Inc., Valencia, CA, USA). Sequencing was performed on the Applied Biosystems 3730 DNA analyzer with POP-7 polymer and a 50-cm capillary array (all from Life Technologies, Carlsbad, CA, USA) following the manufacturer’s instructions. Chromatograms were assembled by using Lasergene SeqMan Pro version 8.0.2 (www.dnastar.com). Sequences were aligned with other *V. cholerae* sequences by using ClustalW (www.clustal.org) in MEGA4 software (*17*) and trimmed in-frame for analyses. The full genome of 1 *V. cholerae* strain from Haiti (2010EL-1786 [ATCC BAA-2163]) and partial genomic regions (integrated conjugative elements and cholera toxin phage) of other isolates were sequenced as described (*18*).

## Results

### Isolates

We identified all 149 isolates from Haiti as *V. cholerae*, serogroup O1, serotype Ogawa, biotype El Tor, containing species-specific genes *ompW* and *toxR*. All contained the cholera toxin gene *ctxAB*. All isolates from countries other than Haiti selected for comparison were confirmed as *V. cholerae*, serogroup O1, biotype El Tor, *ctxAB* positive. Serotypes varied among geographic regions (Table 2).

**Table 2:** Serotype distribution among *Vibrio cholerae* isolates collected during 1991–2011

Location	No. strains typed	Serotype	
		Ogawa	Inaba
Haiti	149	149	0
Afghanistan	2	2	0
Cameroon	1	1	0
Djibouti	2	0	2
Ethiopia	1	0	1
India	6	2	4
Kenya	14	0	14
Nepal	1	1	0
Nigeria	2	2	0
Oman	1	0	1
Pakistan	6	5	1
Peru	2	1	1
Somalia	2	0	2
South Africa	2	2	0
Sri Lanka	1	0	1
Sudan	5	0	5
Togo	2	2	0
US Gulf Coast	1	0	1
Total	200	167	33

### Antimicrobial Drug–Susceptibility

Disk-diffusion testing demonstrated that all strains from Haiti were resistant to furazolidone, nalidixic acid, streptomycin, sulfisoxazole, and trimethoprim/sulfamethoxazole. Strains were susceptible to tetracycline and either susceptible or intermediately susceptible to ampicillin and chloramphenicol. Broth microdilution testing of the isolates from Haiti showed similar results (furazolidone was not tested by broth microdilution) plus decreased susceptibility to ciprofloxacin (MIC range 0.25–1.0 μg/mL). These isolates were susceptible to ampicillin, azithromycin, and chloramphenicol, except for 2 isolates that gave intermediate results for chloramphenicol. Antimicrobial drug susceptibility among strains tested from countries other than Haiti varied; 19 showed the same antimicrobial drug resistance as the Haiti outbreak strains, including isolates from Nepal, Cameroon, South Africa, Oman, and India. Two isolates were similar to the strains from Haiti, but they did not display decreased susceptibility to ciprofloxacin. Twenty-seven isolates from Africa and the Middle East showed the outbreak resistance pattern, but they were susceptible to nalidixic acid and fully susceptible to ciprofloxacin. Two isolates from Peru and the isolate from the US Gulf Coast were susceptible to all antimicrobial drugs tested.

### PFGE Genotypes

Two-enzyme PFGE analyses with *Sfi*I and *Not*I identified a predominant Haiti outbreak pattern combination, KZGS12.0088/KZGN11.0092, in 123 (82.6%) of 149 isolates tested (Table 3). This primary pattern combination was detected in isolates from all 10 departments in Haiti, in isolates from the Dominican Republic, and in isolates from travelers returning to the United States from Hispaniola (data not shown). There were 10 PFGE pattern variations, defined as >1 band difference in number or size from the primary pattern (Figure; Table 3). Variant patterns were detected in isolates from 8 Haiti departments. Of the 10 variant 2-enzyme combinations in the 10 PFGE patterns, the most common was KZGS12.0089/KZGN11.0092, which was found in 8 (5.4%) of 149 isolates. Four new *Not*I and 5 new *Sfi*I restriction patterns and 9 new *Sfi*I/*Not*I PFGE pattern combinations were seen among isolates from this outbreak in Haiti (Table 3).

**Table 3:** *Sfi*I and *Not*I PFGE patterns among toxigenic *Vibrio cholerae* isolates from Haiti, by department and collection date, 2010–2011*

PFGE types	No. (%) isolates										
	All	2010 Oct 26	2010 Nov 26	2011 Jan 27	2011 Feb 9	Artibonite only			West Department only		
						2010 Oct 26	2010 Nov 26		2010 Nov 26	2011 Jan 27	2011 Feb 9
*Sfi*I types											
KZGS12.0088	130 (87.2)	16 (100)	76 (82.6)	27 (90.0)	11 (100)	16 (100)	16 (84.2)		35 (81.4)	21 (87.5)	8 (100)
KZGS12.0089	8 (5.4)	0	8 (8.7)	0	0	0	2 (10.5)		3 (7.0)	0	0
KZGS12.0097†	7 (4.7)	0	4 (4.3)	3 (10.0)	0	0	0		3 (7.0)	3 (12.5)	0
KZGS12.0063†	1 (0.7)	0	1 (1.1)	0	0	0	0		0	0	0
KZGS12.0158†	1 (0.7)	0	1 (1.1)	0	0	0	1 (5.3)		0	0	0
KZGS12.0159†	1 (0.7)	0	1 (1.1)	0	0	0	0		1 (2.3)	0	0
KZGS12.0160†	1 (0.7)	0	1 (1.1)	0	0	0	0		1 (2.3)	0	0
*Not*I types											
KZGN11.0092	137 (91.9)	15 (93.8)	85 (92.4)	26 (86.7)	11 (100)	15 (93.8)	19 (100)		36 (83.7)	21 (87.5)	8 (100)
KZGN11.0034†	5 (3.4)	1 (6.3)	4 (4.3)	0	0	1 (6.3)	0		4 (9.3)	0	0
KZGN11.0142†	4 (2.7)	0	0	4 (13.3)	0	0	0		0	3 (12.5)	0
KZGN11.0124†	2 (1.3)	0	2 (2.2)	0	0	0	0		2 (4.7)	0	0
KZGN11.0134†	1 (0.7)	0	1 (1.1)	0	0	0	0		1 (2.3)	0	0
*Sfi*I/*Not*I combinations											
KZGS12.0088/KZGN11.0092	123 (82.6)	15 (93.8)	73 (79.3)	24 (80.0)	11 (100)	15 (93.8)	16 (84.2)		32 (74.4)	19 (79.2)	8 (100)
KZGS12.0089/KZGN11.0092	8 (5.4)	0	8 (8.7)	0	0	0	2 (10.5)		3 (7.0)	0	0
KZGS12.0097/KZGN11.0092†	4 (2.7)	0	2 (2.2)	2 (6.7)	0	0	0		1 (2.3)	2 (8.3)	0
KZGS12.0088/KZGN11.0142†	3 (2.0)	0	0	3 (10.0)	0	0	0		0	2 (8.3)	0
KZGS12.0088/KZGN11.0034†	4 (2.7)	1 (6.3)	3 (3.3)	0	0	1 (6.3)	0		3 (7.0)	0	0
KZGS12.0097/KZGN11.0124†	2 (1.3)	0	2 (2.2)	0	0	0	0		2 (4.7)	0	0
KZGS12.0063/KZGN11.0092†	1 (0.7)	0	1 (1.1)	0	0	0	0		0	0	0
KZGS12.0097/KZGN11.0142†	1 (0.7)	0	0	1 (3.3)	0	0	0		0	1 (4.2)	0
KZGS12.0158/KZGN11.0092†	1 (0.7)	0	1 (1.1)	0	0	0	1 (5.3)		0	0	0
KZGS12.0159/KZGN11.0034†	1 (0.7)	0	1 (1.1)	0	0	0	0		1 (2.3)	0	0
KZGS12.0160/KZGN11.0134†	1 (0.7)	0	1 (1.1)	0	0	0	0		1 (2.3)	0	0

*PFGE, pulsed-field gel electrophoresis. †Unique PFGE patterns first seen in this outbreak.

**Figure:**
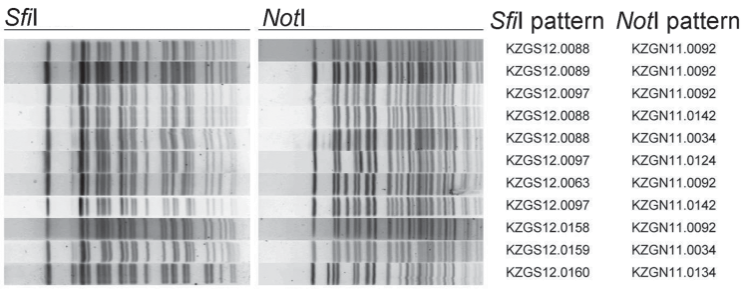
Pulsed-field gel electrophoresis patterns for *Vibrio cholerae* isolates from Haiti, 2010–2011.

All 16 initial isolates (received October 26, 2010) were from Artibonite Department, the source of the outbreak in Haiti, and demonstrated little PFGE diversity. In contrast, 76 additional isolates (received on November 26, 2010) represented all 10 departments and demonstrated substantial variation, including PFGE types never observed (Table 3). The isolates received on January 27, 2011, and February 6, 2011, were primarily from West Department (including Port-au-Prince) and thus did not enable continuing analysis of isolates from other departments. However, enough isolates were available to roughly compare PFGE pattern distributions in 2 departments over time: Artibonite (16 isolates received October 26, 2010, and 19 isolates received November 26, 2010) and West (43 isolates received November 26, 2010, another 24 isolates received January 27, 2011, and 8 isolates received February 6, 2011) (Table 3). In neither department was there an unambiguous trend toward more diversity over time, but the diversity already present in the November 26, 2010, West Department isolates is striking.

Isolates (n = 17) from Afghanistan, Cameroon, India, Nepal, Oman, Pakistan, and South Africa shared the primary PFGE pattern of isolates from Haiti (Table 4). An isolate from South Africa, which was obtained from an outbreak possibly related to the Zimbabwe outbreak in 2009 (*19*), had the most common Haiti variant (KGZS.0089/KGZN11.0092) (Table 4).

**Table 4:** *Vibrio cholerae* isolates from various countries with the *Sfi*I/*Not*I PFGE pattern combinations most commonly found in outbreak isolates obtained from Haiti in 2010–2011*

Location	No. isolates	*Sfi*I/*Not*I PFGE pattern, no. (%)	
		KZGS12.0088/ KZGN11.0092	KZGS12.0089/ KZGN11.0092
Haiti	149	123 (82.6)	8 (5.4)
Afghanistan	2	2 (100)	0
Cameroon	1	1 (100)	0
Djibouti	2	0	0
Ethiopia	1	0	0
India	6	6 (100)	0
Kenya	14	0	0
Nepal	1	1 (100)	0
Nigeria	2	0	0
Oman	1	1 (100)	0
Pakistan	6	6 (100)	0
Peru	2	0	0
Somalia	2	0	0
South Africa	2	1 (50.0)	1 (50.0)
Sri Lanka	1	0	0
Sudan	5	0	0
Togo	2	0	0
US Gulf Coast	1	0	0
Total	200	141	9

*Isolates were collected during 1991–2011. PFGE, pulsed-field gel electrophoresis.

Of 25 cultures with colonies having hemolytic and nonhemolytic phenotypes, only 3 displayed nonidentical PFGE patterns. There was no apparent association of PFGE pattern with hemolysis; 20 (80%) of 25 hemolytic and 22 (88%) of 25 nonhemolytic isolates had the main combination pattern seen in isolates from Haiti. One colony from inside the chloramphenicol zone of inhibition also displayed a variant pattern (data not shown).

### Virulence and Species-specific Genes, PCR Results, and Sequences

The sequencing results for *ctxB* and *tcpA* and PCR results for *rstR* and the VC2346 gene are shown in Table 5. We sequenced the complete 1,148 bp of the *ctxAB* operon for 107 strains. The *ctxB* gene sequence of all 56 isolates from Haiti that we tested matched the B-7 allele first seen in an outbreak in 2008 in Orissa, India (*20*) (Table 5). The B-7 allele was also contained in 1 isolate from Cameroon, 3 of 6 from India, and 1 from Nepal. The B-7 allele has a single-nucleotide polymorphism (SNP) at nt 58 relative to the classical B-1 allele (“genotype 1” [*21*]), resulting in substitution of asparagine for histidine at aa 20, which is adjacent to the B subunit proteolytic cleavage site between aa 21 and aa 22. The remaining isolates from non-Haiti locations carried the B-1 allele, with the exception of the strains from Peru, which carried the B-3 allele (genotype 3, reported by Olsvik et al. [*21*]) that historically has been associated with the El Tor biotype.

**Table 5:** Sequencing and PCR results for *Vibrio cholerae* isolates collected during 1991–2011*

Location of origin	Sequencing results					PCR results			
	No. sequenced for *ctxB*	*ctxB* allele	No. sequenced for *tcpA*	*tcpA* allele		No. tested for *rstR*	*rstR* allele	No. tested for VC2346 gene	VC2346 gene
Haiti	56	B-7	56	ET*^CIRS^*		56	ET	56	Pos
Afghanistan	2	B-1	2	ET, ET*^CIRS^*		2	ET	2	Pos
Cameroon†	1	B-7	1	ET*^CIRS^*		1	ET	1	Pos
Djibouti	2	B-1	2	ET		2	ET	2	Pos
Ethiopia	1	B-1	1	ET		1	ET	1	Pos
India†	6	B-1 (3), B-7 (3)	6	ET*^CIRS^*		6	ET	6	Pos
Kenya	14	B-1	14	ET		2	ET	14	Pos
Nepal†	1	B-7	1	ET*^CIRS^*		1	ET	1	Pos
Nigeria	2	B-1	2	ET, ET*^CIRS^*		2	ET	2	Pos
Oman	1	B-1	1	ET		1	ET	1	Pos
Pakistan	6	B-1	6	ET*^CIRS^*		6	ET	6	Pos
Peru	2	B-3	2	ET		2	ET	2	Pos
Somalia	2	B-1	2	ET		2	ET	2	Pos
South Africa	2	B-1	2	ET*^CIRS^*		2	ET	2	Pos
Sri Lanka	1	B-1	1	ET*^CIRS^*		1	ET	1	Pos
Sudan	5	B-1	5	ET		2	ET	2	Pos
Togo	2	B-1	2	ET		2	ET	2	Pos
US Gulf Coast	1	B-1	1	ET		1	Classic	1	Neg
Total	107		107			92		104	

*ctxB, cholera toxin subunit B; *tcpA*, biotyping gene; *rstR*, biotype-specific repeat sequence transcriptional regulator; Pos, positive; Neg, negative. †By pulsed-field gel electrophoresis and *ctxB* and *tcpA* sequencing, the isolates from Cameroon and Nepal and 3 isolates from India are most closely related to the isolates from Haiti.

A 1,234-bp sequence, which included 675 bp of the entire *tcpA* gene, matched sequences from CIRS 101, a *V. cholerae* serogroup O1, biotype El Tor strain (*22*) that has an integrated El Tor type phage (CTXΦ)^El Tor^ but expresses the classical *ctxB-*1 allele. This combination is considered to be an altered or atypical El Tor. The *tcpA* sequence of CIRS 101 has a novel SNP at nt position 266 (aa 89), which differentiates it from typical El Tor strains (*22*). All 56 tested isolates from Haiti had this novel SNP in the *tcpA* sequence, which we have designated the *tcpET*^CIRS^ allele. Isolates from Africa and southern Asia also had the *tcpET*^CIRS^ allele (Table 5).

The *rstR*^El Tor^ allele was found in all isolates tested except the strain from the Gulf Coast of the United States. All isolates tested, except the strain isolate from the US Gulf Coast, contained the VC2346 gene and are therefore confirmed as seventh pandemic *V. cholerae* (*11*). As shown by whole genome sequencing, isolates with the PFGE pattern KGZS12.0088/KGZN11.0092 (containing the *ctxB*-7/*tcpET*^CIRS^ alleles from Cameroon, India, and Nepal) are most closely related to the strain from Haiti. Details of whole genome sequencing are provided elsewhere (*18*).

## Discussion

We characterized the strains in this study by using basic phenotypic markers and genotypic tests that in other studies have been shown to be useful for characterizing *V. cholerae* outbreak strains. Epidemic cholera strains cause human illness by expression of specific genes that enable *V. cholerae* to exist in the environment long enough to be ingested, overcome host immunity, colonize the intestinal tract, and produce cholera toxin in the host (*13**,**23**–**25*). The horizontal acquisition of genes and expression of the transcriptional co-regulated pilus (located on *Vibrio* pathogenicity island 1) and CTXAB (carried on CTXΦ) determine primary virulence (*9**,**23**,**24*). CTXΦ has 2 regions, a core that includes *ctxAB* and the RS2 region that carries phage replication genes, such as *rstR*. Allelic variations in *ctxB*, *tcpA*, and *rstR* can be useful markers to characterize CTXФ types and track cholera strains. Whereas nucleotide sequence analysis of the acquired virulence genes *ctxAB*, *rstR*, and *tcpA* differentiates among *V. cholerae* O1, the VC2346 gene is part of the nontransferable genomic backbone that identifies seventh pandemic *V. cholerae* O1 El Tor strains currently in circulation.

The adaptability of *V. cholerae* as a pathogen is facilitated by extensive genetic diversity driven by acquisition and recombination of various genetic elements. Community defense mechanisms, mediated by cellular signaling, such as quorum sensing in biofilms, gives the species additional resiliency against changing environmental conditions (*26*). All *V. cholerae* have these attributes, but only O1 and O139 serogroups currently have pandemic potential. The El Tor biotype was first identified 100 years ago, and 50 years ago it emerged from Indonesia to begin the ongoing seventh pandemic, displacing the classical biotype of the fifth and sixth pandemics because of its superior ability to survive in the environment, increased frequency of asymptomatic carriers, and more efficient transmissibility (*27*).

Genes encoding hemolysis, such as *hlyA*, may be a virulence factor in some *Vibrio* spp., and in the past, hemolysis patterns were used to distinguish biotypes (*28*). Classical biotype strains carry deletion mutations in the *hlyA* locus, and many El Tor strains produce no detectable hemolysis on blood agar plates. Our findings confirm observations that hemolysis is not a reliable marker for strain discrimination. Of interest, hemolytic colonies were often observed within a streak predominated by nonhemolytic colonies.

The predominant KZGS12.0088/KZGN11.0092 *Sfi*I/*Not*I PFGE pattern in the strains from Haiti is a relatively new subtype that was first seen in 2005 in the PulseNet USA database in isolates from travelers returning from India. Although the isolates from Haiti show diversity in their PFGE subtyping patterns, the constancy of the main pattern coupled with virulence genotyping results indicate a high clonality of the outbreak strains, which is consistent with a point-source introduction. Such PFGE diversity, similar to what we observed, has been noticed during outbreaks in the Bengal region (T. Ramamurthy, pers. comm.). This primary KZGS12.0088/KZGN11.0092 pattern and its close variant, KZGS12.0089/KZGN11.0092, were found in strains from Afghanistan, Cameroon, India, Nepal, Oman, Pakistan, and South Africa. Among the strains from Haiti, we identified 4 new *Not*I restriction patterns, 5 new *Sfi*I patterns, and 9 new *Sfi*I/*Not*I combinations, a finding suggestive of continuing evolution of the outbreak strain. The PFGE pattern combination KZGS12.0019/KZGN.0092 was commonly seen in serotype Inaba strains originating from East Africa and the Middle East and was recently seen in isolates from Togo; in this study, the pattern was associated with the *ctxB-*1 allele.

Although the strains from Haiti are genetically an El Tor biotype, they contain the classical *ctxB-*7 allele. This allele was first identified in 2007 in strains from an outbreak in Orissa, India (*20*). The appearance of a classical *ctxB* gene in El Tor strains is not unprecedented. In the early 2000s, hybrid El Tor strains emerged carrying CTXΦ^Classical^ with the *ctxB* gene of the classical biotype, which is thought to cause more severe clinical disease (*29*). These hybrid strains most likely arose through horizontal transmission of CTXΦ^Classical^, and the resulting genotypes with the classical *ctxB-*1 allele have spread to Asia and Africa. In this study, we show that *V. cholerae* carrying the *ctxB-*7 allele are also disseminating globally.

The transcriptional co-regulated pilus serves a dual role as the major intestinal colonization factor and CTXΦ receptor. All tested isolates from Haiti had *tcpA* sequences with an SNP at nt 266 (*tcpET*^CIRS^), an allele previously reported in strain CIRS 101 from Bangladesh (*22*), which is an El Tor biotype that produces a classical toxin yet carries CTXΦ^El Tor^; this SNP produces a distinct allele that distinguishes it from classical and typical El Tor. In the present study, isolates from Haiti, Africa, and southern Asia carried *tcpET*^CIRS^ (Table 5). Our results show that the *tcpET*^CIRS^ allele is also spreading globally, although not in tandem with the *ctxB-*7 allele because the *tcpET*^CIRS^ allele was also found in isolates with the *ctxB-*1 allele from Afghanistan, India, Pakistan, South Africa, and Sri Lanka (Table 5). Our findings agree with those from a recent study suggesting a close relationship between 2 isolates from Haiti and isolates from Southeast Asia (*30*). However, we also observed a relationship between isolates from Haiti and Africa.

The finding of these *ctxB-*7*/tcpET*^CIRS^ isolates in Cameroon, India, Nepal, and now Hispaniola is not surprising, given the ease of international travel; we are unable to identify the origin of the Haiti strains because of geographic and temporal limitations in our culture collection. In particular, our findings do not rule out the presence of *ctxB-*7/*tcpET*^CIRS^ isolates in countries not represented in our collection. Whole-genome sequencing results confirmed the genetic relationship of these isolates from Haiti, Cameroon, India, and Nepal (*18*). More extensive whole-genome sequencing studies and other subtyping methods, such as multiple-locus variable-number tandem repeat analysis, hold promise for providing a better understanding of the relationships between isolates.

The strain from Haiti is distinct from the isolate from the US Gulf Coast. The isolate from the Gulf Coast was characterized as KZGS12.0055/KZGN11.0029, *ctxB-*1, *tcpET*, *rstR*^Classical^ and negative for VC2346. The isolate from Haiti is also not related to the isolates from Peru from the 1991 Latin America outbreaks, which were characterized as KZGS12.0114/KZGN11.0033, *ctxB-*3, *tcpET*, and *rstR*^El Tor^.

The strain from Haiti has the core characteristics of the seventh pandemic El Tor clone. Our findings support the widespread observation that the typical El Tor strain, which started the seventh pandemic, is gradually being replaced by El Tor isolates with classical cholera toxin subunits.

The isolates from Haiti and those from other regions displayed a consistent resistance phenotype, with resistance to the clinically relevant antimicrobial drugs trimethoprim/sulfamethoxazole and sulfisoxazole but susceptibility to other primary antimicrobial drug options, including doxycycline and azithromycin. A discussion of integrating conjugative and other mobile genetic elements that can potentially mediate transfer of antimicrobial drug resistance is provided elsewhere in this issue (*31*). Development of additional antimicrobial drug resistance, particularly to doxycycline and macrolides, remains a serious clinical threat, and Laboratoire National de Santé Publique and CDC continue to monitor for the emergence of such resistance.

The strains from Haiti are fully virulent and contain all the genes necessary for orchestrating the expression of *Vibrio* spp. virulence factors. These strain characteristics, coupled with the sudden and explosive course of the 2010 outbreak, are consistent with an introduction of this strain into a vulnerable population at a single point in time.

The adaptive immunity of the local population as well as climate variations will further drive bacterial evolution; for example, it will not be surprising to observe a gradual switch over time from the Ogawa to the Inaba serotype as population immunity to Ogawa rises, as has been reported during several outbreaks (*23*). The primary PFGE patterns continue to diverge as the infections continue, likely reflecting interactions with the host immune system and between environmental and epidemic populations of bacteria networking in complex ways. Regardless, rapid diagnosis and continuing public health control of the current outbreak in Haiti as well as future outbreaks is paramount for limiting sickness and death, and intensive studies using a variety of basic science, diagnostic, and epidemiologic tools will remain useful for reducing the overall global impact of cholera.
